# Integrating community perspectives to improve survey completion rates in public health research by refining controversial survey elements

**DOI:** 10.1017/cts.2025.80

**Published:** 2025-04-28

**Authors:** Arleth A. Escoto, Angel Lomeli, Maria Linda Burola, Breanna Reyes, Ana Perez-Portillo, Scarlet Flores, Kayleigh Kornher, Norma Porras, Ariel Cohen, Linda Salgin, Borsika A. Rabin, Louise C. Laurent, Marva Seifert, Nicole A. Stadnick

**Affiliations:** 1 Department of Obstetrics, Gynecology and Reproductive Sciences, University of California, San Diego, La Jolla, CA, USA; 2 Department of Research and Health Promotion, San Ysidro Health, CA, USA; 3 San Diego State University/University of California San Diego Joint Doctoral Program in Public Health, San Diego, CA, USA; 4 Herbert Wertheim School of Public Health and Human Longevity Science, University of California, San Diego, La Jolla, CA, USA; 5 Altman Clinical and Translational Research Institute Dissemination and Implementation Science Center, University of California, San Diego, La Jolla, CA, USA; 6 Department of Medicine, University of California, San Diego, La Jolla, CA, USA; 7 Department of Psychiatry, University of California, San Diego, La Jolla, CA, USA; 8 Child and Adolescent Services Research Center, San Diego, CA, USA

**Keywords:** Community engagement, common data elements, public health, National Institutes of Health research, underserved communities

## Abstract

**Background:**

Many factors can impact survey completion rates, including survey length, sensitivity of the topics addressed, and clarity of wording. This study used cognitive interviews (CIs), a methodological tool that can aid in developing and refining elements for multi-faceted assessments, and previous survey response patterns to refine, streamline, and increase response rates of RADx-UP Common Data Elements (CDEs) for survey/questionnaire use.

**Methods:**

Ten previously enrolled CO-CREATE study participants were interviewed between May–June 2023. Interviewees identified CDEs that were “confusing, uncomfortable, and/or not applicable,” along with their reasoning. Interview data were analyzed using a rapid qualitative analytic approach, resulting in a summary matrix categorized by language. For further contextualization, CDE response rates were calculated for the 9147 surveys administered during the CO-CREATE study (May 2021–March 2023) and compared against their survey position.

**Results:**

Of the 94 CDEs evaluated in the CIs, 20 (21.3%) were flagged by one or more interviewees. Nine (9.6%) English while fourteen (14.9%) Spanish CDEs were flagged by interviewees, with some overlap. Also, CDE response rates differed according to position in the survey, with lower response rates for questions positioned later in the survey. Following review by the research team and the RADx-UP program, 10 English and 15 Spanish were revised, and seven were removed in both languages in the final survey.

**Conclusion:**

Our findings underscore the importance of integrating community member perspectives to enhance the relevance and clarity of assessment instruments, optimizing the impact of public health research among underrepresented populations.

## Introduction

Common data elements (CDEs) are precisely defined questions and corresponding response options that are used across studies to standardize data collection [1]. Although CDEs were first developed for National Cancer Institute clinical trials in 1999 [2], their use has continued to grow, and is encouraged for research projects funded by the National Institutes of Health (NIH) 2023 Policy for Data Management and Sharing [3]. The advantages of CDEs, contributing to their increasing adoption by funders and researchers, include a uniform data collection infrastructure that streamlines data collection and sharing across studies [4–6]. In addition, access to a centralized database of CDEs through data harmonization establishes a shared evidence base for the research community, which may advance research efforts that include underrepresented groups and individuals with rare or understudied health conditions [7]. CDEs improve system interoperability and enable researchers to combine and analyze data more efficiently across studies, increasing the size and diversity of populations included in meta-analyses [8,9].

Despite these advantages, there are barriers and drawbacks to the use of CDEs. These include the resources and infrastructure, often substantial, needed to collect, report, and share CDEs, all of which are necessary to maximize their value [6]. Another critical consideration is the impact on research participants when collecting survey and questionnaire data using CDEs [10]. When CDEs are used in research across populations and settings, cultural appropriateness, health and reading literacy, and meaningfulness of the items are essential to consider. Data elements that participants view as unnecessary, irrelevant, or unduly time-consuming [11] may negatively impact data integrity and trust between participants and researchers [12]. This may have long-term, negative impacts on research participation and inclusiveness of participants from diverse backgrounds and underserved communities which has been increasingly highlighted as a concern that may exacerbate health disparities for communities of greatest need [13,14].

Another significant consideration in the use of CDEs is survey fatigue among research participants. Survey fatigue (also referred to as respondent fatigue) is the phenomenon where respondents become tired, disengaged, or less responsive due to the length or frequency of surveys they are asked to complete [15–17]. Survey fatigue can lead to “satisficing,” which is the tendency to seek quick answers to complete the survey rather than providing thoughtful and accurate responses, compromising the quality of the data collected [18,19]. With the proliferation of survey data collection efforts across various studies, survey length, brevity, clarity, and prioritizing the inclusion of culturally relevant and meaningful CDEs are essential to consider throughout the design process of survey-based studies.

Emerging research demonstrates the value of community-engaged approaches to mitigate disadvantages and facilitate the use of CDEs in public health research [20,21]. For instance, the Value-Based Framework has illustrated the importance of value exploration as a central first step for community-centered study designs for public health research initiatives [22]. This approach has facilitated the co-creation of a “research identity” among community members, thereby increasing community ownership and engagement [22]. Furthermore, the Community-Centered Evidence-Based Practice (CCEBP) approach has offered an enhanced evidence-based practice (EBP) model for community-based organizations working alongside Latina/o and other diverse communities [23]. This approach prioritizes community expertise to protect communities against indiscriminate research practices that are harmful to the needs of Latino/communities and other underrepresented groups [23]. Key principles for community-engaged research have been established to reimagine community engagement by improving resilience of partnerships, ensuring inclusivity of community voices throughout the design process, and ultimately creating an equitable future for the most vulnerable groups in our society [24,25].

Federal research funders have used community engagement strategies to design and improve data collection procedures, including CDEs. The US Office of Minority Health developed a pilot uniform data set to serve as a primary data collection system that is shareable across programs for all grants and standardized agreements funded within the U.S. Department of Health and Human Services [26]. All content was developed through a formative research process involving an advisory panel, focus groups, interviews, site visits, and pilot testing aimed at identifying appropriate data elements [26]. While structural and practical issues emerged throughout the implementation process, these standardized efforts improved data monitoring across widely disparate projects, contributing to a more meaningful data collection system for Community-Based Organizations and racial/ethnic minority populations.

A second example that is specific to the current study is the process used to develop Rapid Acceleration of Diagnostics-Underserved Populations (RADx-UP) CDEs. RADx-UP is an NIH-funded consortium of community-engaged research projects aiming to increase COVID-19 testing access in underserved populations [27]. The RADx-UP Coordination and Data Collection Center was led by the Duke Clinical Research Institute and engaged 69 community and academic RADx-UP teams to select, refine, and standardize CDEs. While this was a critical step to facilitate acceptability, appropriateness, and data integrity of CDEs, challenges emerged related to missing data and persisting concerns about relevance to underserved communities asked to participate in this research.

Cognitive interviewing, a qualitative method that collects feedback throughout the measure development process, is particularly useful for developing multi-level assessment procedures that capture discrepant partner and community viewpoints [28]. This approach, as we present in this study, can facilitate a shared understanding between partners and implementation measure developers, facilitating a common language in the field. This approach was motivated by concerns from study participants and members of the study’s Community and Scientific Advisory Board (CSAB) about the relevance and value of CDE items. This multi-method process presented in this study fills a critical gap in the literature, demonstrating the vital importance of community-engaged pragmatic approaches for the evaluation of evidence-based intervention measures. The study’s objectives are to report the use of survey and cognitive interview results to identify potentially problematic CDEs and to use a community-engaged, pragmatic approach to refine those CDEs.

## Methods

### Study design

This study is part of a larger program of research conducted through the NIH-funded RADx-UP initiative. Our local site has serially conducted two RADx-UP studies: first, the Community-driven Optimization of COVID-19 testing to Reach and Engage Underserved Areas for Testing Equity (CO-CREATE) study was funded by a RADx-UP Phase 1 grant and was conducted from May 2021–March 2023; next, the Community-engaged Optimization of COVID-19 Rapid Evaluation and Testing Experiences (CO-CREATE-Ex) study was funded by a RADx-UP Phase 3 grant and was launched September 2023 [29]. To gain in-depth feedback about the CO-CREATE CDEs used in Phase 1 to refine, streamline, and increase response rates for the CO-CREATE-Ex Phase 3 study, our team conducted cognitive interviews with community members who participated in Phase 1 research activities and analyzed CDE completion rates for the Phase 1 survey. Our efforts were motivated by concerns from study participants and members of the study’s Community and Scientific Advisory Board about the relevance and value of CDE items. All study procedures were approved by the Institutional Review Boards of the University of California San Diego and the partnering FQHC.

### Participants

**Figure 1.**
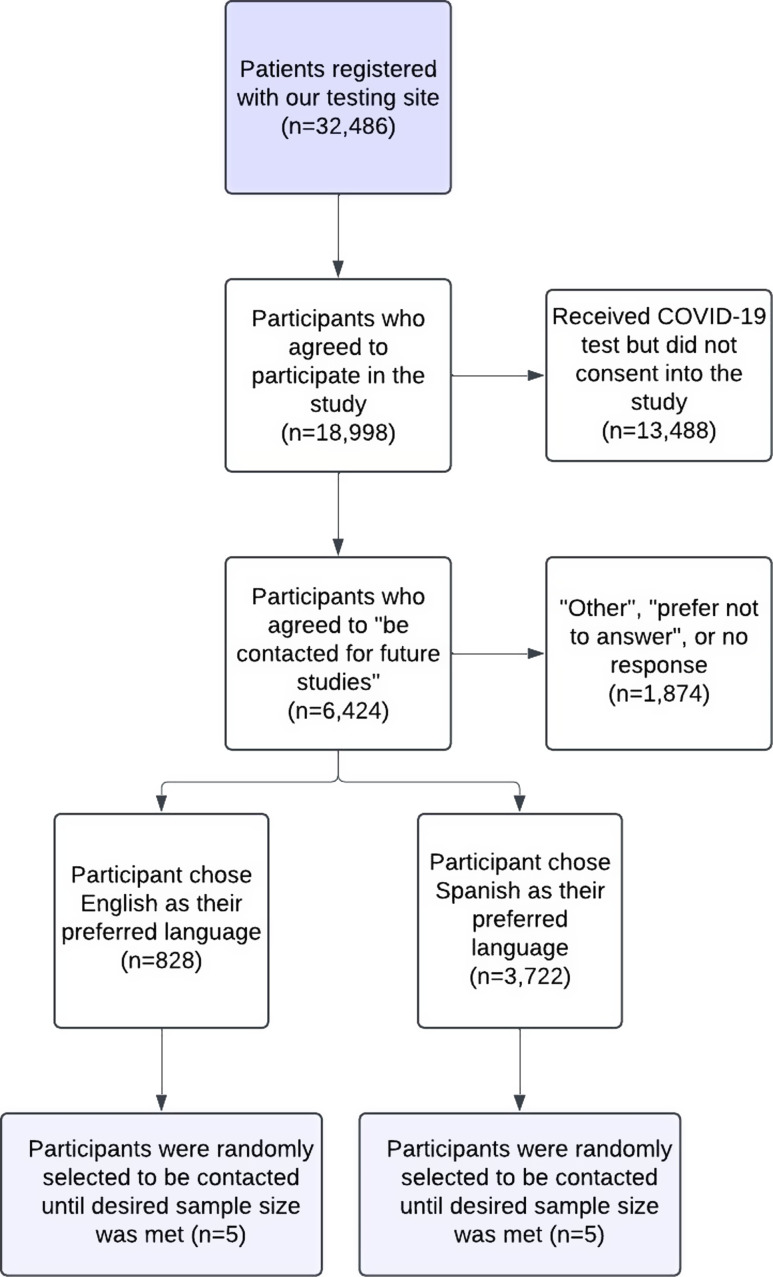
Flowchart of sampling method for study participant determination measures.

Ten previously enrolled CO-CREATE study participants (five English-speaking and five Spanish-speaking) were invited to participate in a cognitive interview. Inclusion criteria for participant selection were: 1) must have completed the CO-CREATE Phase 1 survey that included NIH CDEs (see Measures below); 2) agreed to be contacted for future studies on the informed consent that they signed to complete the Phase 1 survey; 3) tested with the CO-CREATE program at least 3 times; and 4) were at least 18 years of age at the time of contact. Participants were compensated USD50 for their participation. Fig. 1 describes the sampling method and final sample size for those who participated as cognitive interviewees.

#### Phase 1 CO-CREATE survey

The Phase 1 CO-CREATE Survey included a total of 198 items, 167 were RADx-UP version 1.2 CDEs, 12 were CO-CREATE-specific questions, and 19 were registration (consent and contact information) questions. The Phase 1 CO-CREATE Survey was structured into 13 sub-sections, with data collected encompassing information about demographics, health status, vaccination status, exposure risk, testing history, household details, financial situation, employment status, and lifestyle habits such as alcohol and tobacco use. Questions were presented in the same sequence to all CO-CREATE participants. The survey took participants approximately 25–30 minutes to complete.

#### Phase 3 CO-CREATE-ex survey

The proposed Phase 3 CO-CREATE Survey included a total of 94 RADx-UP version 1.6 CDEs. The questions encompassed themes such as demographics, household details, employment status, health status, COVID-19 and Long COVID-19 symptoms, and lifestyle habits such as alcohol and tobacco use.

### Procedures

To address the concerns presented by study participants and members of the Community and Scientific Advisory Board (CSAB), individual cognitive interviews were conducted between May 10, 2023, and June 2, 2023. All interviews were led by four trained bilingual research staff. Participants were first asked to review the Phase 3 survey independently and mark any items that were “confusing, make you uncomfortable, and/or do not apply to you.” During this independent review, participants were also asked to write down their reasoning for the questions marked as causing confusion, discomfort, or not applicable. Following independent review, the interviewer asked the participant to verbally explain their reasoning for each item marked as problematic or not applicable. Each interview lasted 45–60 minutes. Interviews were audio-recorded and professionally transcribed by an online service. Eight research staff and the PIs participated in three 1hour meetings to review participant CI results and reach a consensus on refined and revised CDEs.

### Data analysis

The transcribed interview data were analyzed using a rapid qualitative analytic approach [30,31] to identify and contextualize reasons why CDEs were considered confusing, causing discomfort, or not applicable to participants. Specifically, a matrix of summary responses from each question and divided by response language (Spanish or English) in the interview was developed. Clinical research staff and investigators reviewed the matrix of summary responses to either develop revisions to survey questions flagged by participants or remove them from the Phase 3 CO-CREATE-Ex survey. To further contextualize the cognitive interview data, response rate frequencies of Phase 1 survey data were conducted.

The 167 CDEs captured in the Phase 1 survey included data elements that did not require a response from participants, including date stamps captured electronically and headers included for clarification of multi-part questions. Response rate was calculated by dividing the number of responses by the total number of individuals who would have encountered the question based on branching logic. The survey employed branching logic for specific questions, for example, only individuals who marked “yes” in response to the question “Have you ever been tested for COVID-19?” were then asked, “Have you ever tested positive for COVID-19?” Response rates that incorporated branching logic were calculated as the total number of non-responses divided by the total number of individuals who would have had the opportunity to respond to the question. Non-response included “prefer not to answer” and “non-applicable” along with all missing responses. For analysis purposes, questions that were asked of all individuals who completed the survey and were not the result of branching logic were considered “core” questions and remaining questions that were asked of a varying number of individuals were classified as “branching.” Not all questions were included in the original survey and response rates were calculated based only on the total number of surveys completed after the inclusion of additional questions. Free text response questions, after selecting “other,” were excluded from the analysis.

## Results

A total of 10 patient interviews were completed, with 50% of the interviews conducted in Spanish, and the other half conducted in English, per the interviewee’s preferred language. Most interviewees (80%) self-classified as Hispanic/Latino(a), female (70%), and had an average age of 47.6 years. Out of the 94 CDEs (RADx-UP Common Data Elements version 1.6) included in the proposed Phase 3 survey, 20 (21.3%) were flagged as confusing, causing discomfort, and/or not applicable by one or more cognitive interview participants. Nine (9.6%) English CDEs were flagged while fourteen (14.9%) Spanish CDEs were flagged, with overlapping items across languages. After a formative review of CDEs flagged by both participants and research staff, 10 English and 15 Spanish were revised for increased clarity and relevance and 7 were removed in both languages. Cross-language revisions increased the number of modified CDEs than originally flagged for revision. Table 1 reports the item-level response patterns across the 10 interviewees. Supplementary table’s 1 and 2 provide detailed revision and removal information for each flagged question marked in the cognitive interview process.

**Table 1.** Summary of findings of National Institutes of Health common data elements (CDEs) by language for the proposed Phase 3 CO-CREATE-Ex Survey (*n* = 10)

Survey Section Category	Version 1.6 CDEs	CDEs Flagged in English	CDEs Flagged in Spanish	CDEs Retained from Version 1.6					
				English			Spanish		
				*Removed*	*Revised*	*Unchanged*	*Removed*	*Revised*	*Unchanged*
Demographics	12	3	4	0	3	9	0	1	11
Housing, Employment, and Insurance Collection	19	3	3	1	2	16	1	1	17
Work/PPE	3	0	2	0	0	3	0	1	2
Health Status	23	0	4	0	1	22	0	4	19
Disability	7	0	0	0	0	7	0	1	6
COVID-19 Testing and Vaccination	12	1	0	5	1	6	5	1	6
Alcohol/ Tobacco Use	5	0	1	1	2	2	1	1	3
Long COVID	13	2	0	0	1	12	0	5	8
Total	94	9	14	7	10	77	7	15	72
				Total CDEs Retained in New Version			Total CDEs Retained in New Version		
				87			87		

CDE: Common Data Elements.

The research team then examined response rate frequencies from the CO-CREATE Phase 1 survey CDEs. Between May 2021 and March 2023, 9147 participants in the COCREATE study were asked to complete an optional survey comprised of 167 RADx-UP version 1.2 CDEs. Nine date stamp CDEs, 6 header CDEs, and 13 free response CDEs were excluded from the analysis. The response rate was then calculated for the remaining 139 CDEs. The average response rate was 65.5% for the 42 core questions and 70.8% for the 96 branching questions. CDE core question response items with the highest rates of missing response data are included in Table 2, and items with the highest rates of participants endorsing the response option, “I choose not to respond” are also included in Table 2. One of the CDEs, identified by both English and Spanish-speaking interviewees as confusing and/or uncomfortable, also had the second lowest “response” percentage and the highest “I choose not to respond” percentage.

**Table 2.** Five core questions with the lowest overall response rates and the highest “choose not to respond” rates in the Phase 1 CO-CREATE survey

Percent Response	Survey Question	Survey Position
The five **core** questions with the lowest “response” percentage were:		
47.6%	What do you think your personal level of risk is for getting sick from COVID-19?	110
49.7%	Which of the following best represents how you think of yourself at this time?**	17
50.9%	[Do you have any of these symptoms during the past week?] Other [symptoms]	32
57.7%	[Has anyone close to you] died from COVID-19?	108
57.9%	[Has anyone close to you] Become sick from COVID-19?	106
The five **core** questions with the highest “I choose not to respond” percentage were:		
7.5%	Which of the following best represents how you think of yourself at this time?**	17
2.3%	What do you think your personal level of risk is for getting sick from COVID-19?	110
2.0%	Are you of Hispanic, Latino, or Spanish origin?	9
1.4%	[Has anyone close to you] died from COVID-19?	108
1.4%	Do you speak a language other than English at home?	12

** Common Data Elements (CDEs) flagged in both English and Spanish by cognitive interviewees.

**Figure 2.**
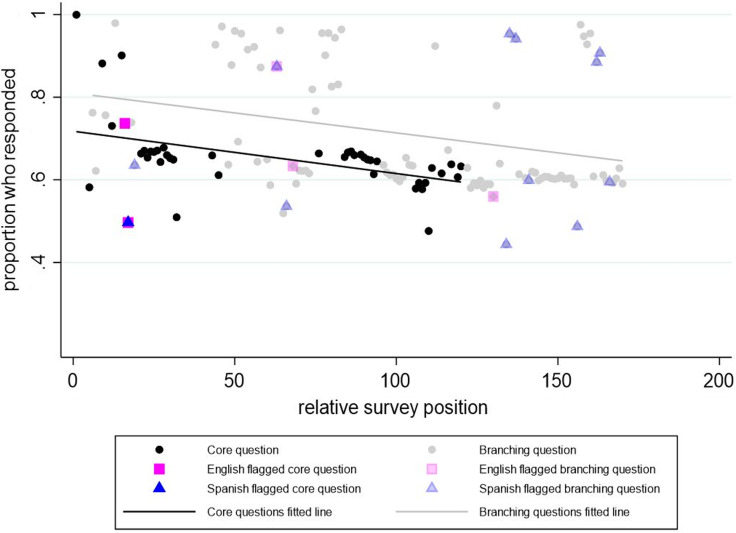
Response rate versus relative survey position in the Phase 1 CO-CREATE Survey. *Note*: The Phase 1 CO-CREATE Survey did not include all the common data elements reviewed in the cognitive interview process.

The response rate for each CDE was compared against its position in the survey, and a linear trendline was drawn separately for both core and branching questions, with an R^2^ of 19.8% and a slope of −0.0010 (*p* value = 0.003) for core questions and an R^2^ of 8.5% and slope of −0.0010 (*p* value = 0.004) for branching questions (see Fig. 2). There is a clear pattern of decreasing response data in later sections of the survey and the rate of response decline is similar between core and branching questions, however for branching questions there was more variation in the response rate and the relative survey position.

CDEs that deviated from the core questions trendline by >0.1 were identified. Seven CDEs reached this threshold, with three having completion rates greater than expected and four having completion rates less than expected compared to their position in the survey. The CDEs completed at rates greater than expected included birthdate, sex assigned at birth, and identification of Hispanic, Latino, or Spanish origin. CDEs completed at rates less than expected included identification of race, sexual orientation, perceived level of self-risk from COVID-19, and identification of other symptoms. Of the four CDEs with lower-than-expected response rates, one CDE related to sexual orientation was also flagged by interviewees.

## Discussion

This report describes a multi-method community-engaged approach to refine NIH CDEs for the CO-CREATE and CO-CREATE-Ex studies. Our efforts were motivated by concerns from study participants and members of the study’s Community and Scientific Advisory Board about the relevance and value of CDE items, as well as our review of survey responses that revealed high rates of missing response data for some survey items. Despite the RADx-UP CDE development process engaging academic-community teams funded by this NIH mechanism [32], our study illustrates the importance of ongoing refining and local tailoring of research protocols that may be needed for public health implementation research.

Based on our review of survey response patterns from the CO-CREATE Phase 1 study, the overall response rate was higher for branching questions, and response rates decreased as position in the survey increased for both the core and branching questions, indicating a possible similar survey completion fatigue. To clarify and revise CDE elements to increase their likelihood of completion and to promote their relevance and value to community participants, 10 individual cognitive interviews with former CO-CREATE Phase 1 participants were conducted. Overlap was identified among CDEs flagged in the CI process and those with outlier response rates. For instance, one CDE related to sexual orientation was identified as having the second lowest response rate percentage, the highest frequency of “I choose not to respond” responses, and was flagged by cognitive interviewees as “uncomfortable” and “confusing.” Previous findings have identified similar controversies with survey questions related to sexual orientation [33,34]. A research report series published by the Office of Survey Methods Research found that sexual orientation and gender identity questions were found most difficult to understand by lesbian, gay, bisexual, transgender respondents and those who found difficulty aligning their self-identity with the response options provided [33]. Furthermore, a study that also used cognitive interviewing methods explored ways adolescents may interpret questions related to sexual orientation and found that questions that measured sexual identity were consistently presented as the most difficult to answer by participants [34]. These findings support the imperative need to develop clear and valid measures related to sexual orientation, among other controversial survey elements, to ensure survey elements can capture meaningful and accurate responses.

Outlier response rates were identified, in both directions, for flagged CDEs (higher and lower than expected response rates) for branching survey questions as survey position increased. In contrast, core questions only showed low response rates as survey position increased. However, it is important to consider that the survey used branching logic for specific questions throughout the survey. This means that only certain questions appear when participants select a response that requires a response to an additional question, which could have contributed to this stark difference. Results from the interviews were reviewed with the research team to determine whether to revise the language of the question or response options or request an exemption to remove the CDE item entirely from subsequent data collection waves. In total, 87 CDE items and responses were retained and, of those retained, 10 English and 15 Spanish were revised. Our research team is currently comparing survey response data and completion rates between the Phase 1 survey and revised Phase 3 survey.

While our study is among the few focused on using a community-engaged approach to revise NIH CDEs, similar processes have been employed in other health research to improve their surveys and questionnaires. For example, a previous study used content validity and cognitive interview methods to evaluate an Illness Perception Questionnaire for African Americans with type 2 diabetes, demonstrating that ensuring cultural appropriateness and clarity of survey questions can significantly enhance the interpretation of questions included in questionnaires used in diverse populations [35]. In addition, another study used cognitive interviews and a quantitative field test to provide evidence for the value of cognitive interview methods as a necessary tool for the survey development process [36]. This approach showed how the early identification of problematic survey elements can provide guidance for the optimization of surveys/questionnaires throughout the development period [36]. These findings highlight the importance of using cognitive interview methodology to optimize survey development for public health research, particularly among underserved and diverse populations. Integrating this process can contribute to enhancement of survey measures, improving the quality of research by ensuring representation of diverse experiences in health research.

### Strengths & limitations

This study has several strengths. The primary strength is the robust, multi-method, and community-centered approach used to identify concerns about CDEs used in surveys and to revise them accordingly. The CO-CREATE and COCREATE-Ex studies are founded on established trust between underserved community members, researchers, and service delivery agents. In response to the concerns and experiences of community members completing surveys, our team engaged the community in clarifying CDE challenges and co-designing solutions that would benefit research advancement and promote community trust. Relatedly, we used an empirical approach to identifying CDE challenges through examination of survey response patterns followed by a qualitative explanatory approach through the cognitive interviews to streamline the CDE revision process. This study also has some limitations. The primary limitation is that our approach is centric to our study’s geographic region and priority communities near the US/Mexico border. Another limitation includes our small sample size, which limits the generalizability of our findings. We also recognize we did not address CDEs that are not survey or questionnaire based. However, the multi-method process described is both a replicable and adaptable process that can be tailored to fit the needs of different study populations and variables included in future studies.

## Conclusion

Our approach underscores the importance of integrating community member perspectives to enhance the relevance and clarity of assessment instruments. In addition, analyzing response patterns of previously used CDEs can provide further evidence of problematic CDEs that have been subsequently flagged during the cognitive interview process. This iterative process can facilitate the refinement of survey instruments, optimizing the value and impact of public health research among underrepresented populations.

## Supporting information

Escoto et al. supplementary material Escoto et al. supplementary material

## Supplementary material

The supplementary material for this article can be found at https://doi.org/10.1017/cts.2025.80.
