# Supplementary material for: Integrating community perspectives to improve survey completion rates in public health research by refining controversial survey elements
Source: J Clin Transl Sci. 2025 Apr 28;9(1):e123. doi: 10.1017/cts.2025.80 (PMC12209967; doi:10.1017/cts.2025.80)
Supplement: Escoto et al. supplementary material [file S2059866125000809sup001.docx]

**Supplementary Material**

**Table 1.** Supplementary table of Common Data Elements (CDEs) revised from items flagged by cognitive interviewees

| **Survey Section Category** | **Original Version (1.6)** | | **Revised Version (1.6)** | | **Flagged?** | |
| --- | --- | --- | --- | --- | --- | --- |
|  | English | Spanish | English | Spanish | English | Spanish |
| **Demographics** | Specify other languages read, understood or spoken at home. | Especifique otros idiomas que se lean, comprendan o hablen en su hogar. |  |  | Yes | Yes |
| **Demographics** | Specify other preferred language. | Especifique otro idioma de preferencia. |  |  |  | Yes |
| **Demographics** | What is the highest level of education you have achieved outside or in the United States? Grades roughly equivalent to years of school. | ¿Cuál es el nivel de educación más alto que ha logrado fuera o en los Estados Unidos? Los grados son aproximadamente equivalentes a los años de educación. | What is the highest level of education you have achieved in any country? |  |  | Yes |
| **Demographics** | What terms best express how you describe your gender identity? | ¿Qué términos expresan mejor cómo describe su identidad de género? | What is your gender identity? |  | Yes |  |
| **Demographics** | Which of the following best represents how you think of yourself at this time? | ¿Cuál de las siguientes opciones representa mejor lo que piensa de sí mismo en este momento? | What best describes your sexual orientation? | ¿Cuál de las siguientes opciones representa mejor cómo se identifica en este momento? | Yes | Yes |
| **Housing, Employment and Insurance Collection** | What best describes the people at your home: | ¿Qué opción describe mejor a su familia en el hogar? | Who lives in your home? |  | Yes |  |
| **Housing, Employment and Insurance Collection** | Are you considered an essential worker? An essential worker is someone who was required to go to work even when stay at home orders were in place | ¿Se considera usted un trabajador esencial? Un trabajador esencial es alguien que estaba obligado a ir a trabajar incluso cuando hay órdenes de quedarse en su hogar |  |  | Yes | Yes |
| **Housing, Employment and Insurance Collection** | Did you lose health coverage because of the COVID-19 pandemic? | ¿Perdió la cobertura médica debido a la pandemia de COVID-19? |  | ¿Perdió la cobertura de salud debido a la pandemia de COVID-19? |  | Yes |
| **Housing, Employment and Insurance Collection** | Getting the health care I need (including for mental health) | Recibir la atención médica que necesito (incluida la salud mental) |  |  | Yes |  |
| **Work/PPE** | In your workplace, do you have access to necessary facilities to wash? | En su lugar de trabajo, ¿Tiene acceso a las instalaciones necesarias para lavarse? |  | En su lugar de trabajo, ¿Tiene acceso a las instalaciones necesarias para lavarse las manos? |  | Yes |
| **Work/PPE** | In your workplace, do you have access to necessary personal protective equipment (PPE)? | En su lugar de trabajo, ¿tiene acceso a equipos de protección individual (EPI)? |  |  |  | Yes |
| **Health Status** | Do you have any of the following conditions? (Select all that apply) | ¿Tiene alguna de las siguientes afecciones? (Marque todas las opciones que correspondan) |  | ¿Tiene alguna de las siguientes condiciones? (Marque todas las opciones que correspondan) |  | Yes |
| **Health Status** | How tall are you without shoes? | ¿Cuál es su altura sin zapatos? |  | ¿Cuál es su estatura sin zapatos? |  | Yes |
| **Health Status** | How much do you weigh without clothes or shoes? If you are currently pregnant, how much do you weigh before your pregnancy? | ¿Cuánto pesa sin ropa ni zapatos? Si está embarazada, ¿cuánto pesaba antes de su embarazo? | How much do you weigh? | ¿Cuánto pesa? |  | Yes |
| **Health Status** | Autoimmune disease | Enfermedad autoinmune |  | Afección de inmunodeficiencia |  | Yes |
| **Covid-19 Testing and Vaccination**  ****** | Why would you/did you NOT get a COVID-19 vaccine? | ¿Por qué NO se vacunaría (vacunó) contra el COVID-19? | Why would you NOT get a COVID-19 vaccine? \| Why would you get a COVID-19 vaccine? | ¿Por qué se vacunaría (vacunó) contra el COVID-19? \| ¿Por qué NO se vacunaría contra el COVID-19? | Yes |  |
| **Long Covid** | Do you know what Long Covid is? | ¿Sabe qué es el COVID persistente? |  | ¿Sabe qué es COVID persistente? | Yes |  |
| **Long Covid**  ******* | Has a doctor diagnosed you with Long Covid? | ¿Un médico le ha diagnosticado COVID persistente? | Long COVID is an illness that people may get after COVID-19. Common signs include fatigue, breathing problems, brain fog, stroke, heart attack, and poor control of blood sugar. | El COVID persistente es una enfermedad que las personas pueden contraer después de tener COVID-19.  Los signos más frecuentes son fatiga, problemas respiratorios, disfunción cognitiva, derrame cerebral, infarto de miocardio y mal control del azúcar en sangre. | Yes |  |

*****Survey element was divided into two questions.***

******Description of Long COVID was added to survey element.***

**Table 2.** Supplementary table of Common Data Elements (CDEs) removed from items flagged by cognitive interviewees

| **Survey Section Category** | **Original Version (1.6)** | | **Flagged?** | |
| --- | --- | --- | --- | --- |
|  | **English** | **Spanish** | **English** | **Spanish** |
| **Alcohol/Tobacco Use** | In your entire life, have you had at least 1 drink of any kind of alcohol, not counting small tastes or sips? | En toda su vida, ¿ha tomado al menos 1 bebida de cualquier tipo de alcohol, sin contar las probaditas o sorbos? |  | Yes |
| **Housing, Employment, and Insurance Collection** | In 2019, what was your total household income before taxes? | En 2019, ¿Cuáles fueron sus ingresos domésticos totales sin los impuestos? |  | Yes |
